# Reflex Symptoms Due to Phimosis

**Published:** 1889-10

**Authors:** 


### REFLEX SYMPTOMS DUE TO PHIMOSIS.

IN THE *Lancet* of April 27th, Grant Langhorne relates the case of a boy, aged five years, who had suffered for two years from a dry, convulsive cough, especially marked at night, and which was at times accompanied by convulsions. Many physicians had treated the case without result. Langhorne himself had treated the child for a long time without discovering the cause of or ameliorating the symptoms, until one time, having been hastily summoned, he found the child lying in convulsions, with difficult breathing, livid face, and dilated pupils. He discovered, accidentally, that the prepuce was very long and completely adherent, and circumcised the boy, when he had the satisfaction of seeing the convulsions, and also the cough, permanently cured.

Such a case is interesting for a double reason: First, as showing how remote the cause may be from the scene of the effects; and, second, as illustrating the need for a more thorough examination of cases in which symptoms persist after ordinary therapeutic measures addressed to their relief. Especially in the case of children, where the question of modesty does not need to be considered, is it desirable to strip the patient so that every part of the surface of the body is visible to the naked eye, and can be thoroughly examined. It is a method which has frequently stood us in good stead, where others, more experienced, have overlooked the cause of the trouble. Only recently we were asked to operate upon a case in which the physician in attendance had diagnosticated an existing phymosis as the *fons et origo mali*. The patient, a child four years old, had, up to two years of age, been intelligent and kindly dispositioned. After this time he became sulky and malicious, requiring to be tied to his bed throughout the day. His mental condition at the time we first saw him was that of the ordinary imbecile, and for the past few months he had been growing slowly worse. Circumcision was made, and to-day (some four months since the operation) the boy has markedly improved, runs outdoors alone, is not malicious, and is becoming mentally improved, as shown by his use of his memory and reason. His age, the short lapse of time since circumcision, and the long duration of his malady, of course make it less easy to judge of his actual mental state, and to prognosticate concerning it, but everything points to a complete and permanent cure. Yet here was a case where eminent physicians had been consulted. Men whose ability and experience we have the best of reasons for respecting, and who had failed to recognize the cause of the trouble. All because the child's clothes had not been removed. The attending physician told us the mother had remarked upon his method of examination, saying "the other doctors never did that."

We recall another case, in which tenotomy had been practised for a talipes equino-varus by a surgeon of fame and experience, in which circumcision effected a thorough and speedy cure. Such cases as these are full of interest and instruction, and demand the careful consideration of every painstaking physician. Especially should surgeons pay heed to reflex phenomena, as they so often depend upon some surgical trouble. To Hilton, perhaps, more than any other one surgeon, do we owe our thanks for the clear clinical descriptions given of this class of cases. Such a work as "Rest and Pain" must have a widespread influence, and induce every cautious surgeon to examine carefully any case presenting reflex symptoms not easily accounted for. We hope that a second Hilton may soon arise to add valuable contributions of a similar nature, and to teach us thoroughness.

As for the condition just considered (phimosis), it usually causes only local inconvenience, but the physician should remember that severe bladder symptoms, simulating those of stone, hematuria, spastic contractions, reflex paralysis, choreic symptoms, cough and general convulsions, as in Langhorne's case, can be dependent upon a contracted prepuce. Until this possibility is recognized and a *thorough* examination made, many cases will go on without being diagnosticated and, therefore, without successful treatment.

